# Dr. S. Weir Mitchell

**Published:** 1914-02

**Authors:** 


### OBITUARIES.

---

*Readers are requested to report promptly the death of all physicians in Western New York, or former residents of this region, or graduates of any medical school in Western New York and to notify the families of the deceased of our desire to publish adequate obituary notices.*

---

Dr. S. Weir Mitchell of Philadelphia died at his home, January 4, 1914. He was born in Philadelphia February 15, 1829, was educated in the local schools and at the University of Pennsylvania and graduated in medicine at Jefferson in 1850. He served as a surgeon of volunteers from 1862 till the close of the Civil War, paying special attention to the nervous results of military traumatisms. Five institutions conferred upon him the degree of LL. D., and he held many positions of honor in the medical profession. It is, in a sense, unfortunate that his popular and general professional reputation as a medical man, depended mainly on his rest cure for neurasthenia and hysteria, since, this, however ingenious and practical, was necessarily only a system involving details of medical art. Having heard his masterly demonstrations at the time when Victor Horsley's experiments as to cerebral localizations were new, we can personally attest his higher claim as a medical scientist and critical clinician in his chosen field of neurology.

Dr. Mitchell is the only instance that occurs to us of a man who gained and maintained distinction both as a medical practitioner and as an author in general literature. Many physicians, like himself, have contributed valuable articles to popular literature, dealing with subjects suggested by professional experience. Many others, especially before the modern development of medical science as an engrossing occupation, united medical with other scientific and philosophic authorship; several literary men of distinction, such as Oliver Wendell Holmes and A. Conan Doyle, have passed from creditable medical practice into authorship along other lines; quite a number of men well known as medical practitioners and students have put forth literary works in limited number, of interest but, so far as we recall, no one else has thus combined medicine and literature, especially in the field of fiction, has kept up an active interest in each, and has won high esteem in each according to accepted standards of criticism in each independently of the other. If this statement is an error, we will welcome correction.

Like many other men of high attainment, Dr. Mitchell, although somewhat handicapped physically in his youth, maintained both physical and intellectual vigor to an advanced age· "Westways," published only a few months ago suggests the age of the author only by the maturity of view and by inference from the obvious personal familiarity with the times. This novel, covering the period from about 1850 to 1865, avoids the typic local color of political centers, Southern plantations, Northern hotbeds of abolition and draws very little upon the easily attractive novelist's stock in trade of the battle field. As in most of his other writings, Dr. Mitchell deals mainly with the American aristocracy—to use a convenient term in spite of inviting criticism—but, in spite of this, he avoids the suspicion of snobbishness and does not alienate the sympathies of the reader. Among his numerous and well known stories, we commend especially to medical readers, the "Autobiography of a Quack" as a more valuable lesson in ethics than the code. One remarkable fact about Dr. Mitchell's authorship is that, in the frequent allusions to medical matters, he almost never applies directly his own medical knowledge. As an author, his viewpoint is that of the intelligent laity, of the time and his references to medicine, in his fiction, are developed as literary material and not as clinical or pathologic. Perhaps this is why he attained distinction in a double way, being able to concentrate as a medical man on professional problems and being distinctively a literary writer so far as his fiction is concerned.

